# Variations of X Chromosome Inactivation Occur in Early Passages of Female Human Embryonic Stem Cells

**DOI:** 10.1371/journal.pone.0011330

**Published:** 2010-06-25

**Authors:** Tamar Dvash, Neta Lavon, Guoping Fan

**Affiliations:** 1 Department of Human Genetics and The Eli and Edythe Broad Center for Regenerative Medicine and Stem Cell Research, David Geffen School of Medicine, University of California Los Angeles, Los Angeles, California, United States of America; 2 The International Stem Cell Research Institute, Cedars Sinai Medical Center, Los Angeles, California, United States of America; Radboud University Nijmegen, Netherlands

## Abstract

X chromosome inactivation (XCI) is a dosage compensation mechanism essential for embryonic development and cell physiology. Human embryonic stem cells (hESCs) derived from inner cell mass (ICM) of blastocyst stage embryos have been used as a model system to understand XCI initiation and maintenance. Previous studies of undifferentiated female hESCs at intermediate passages have shown three possible states of XCI; 1) cells in a pre-XCI state, 2) cells that already exhibit XCI, or 3) cells that never undergo XCI even upon differentiation. In this study, XCI status was assayed in ten female hESC lines between passage 5 and 15 to determine whether XCI variations occur in early passages of hESCs. Our results show that three different states of XCI already exist in the early passages of hESC. In addition, we observe one cell line with skewed XCI and preferential expression of X-linked genes from the paternal allele, while another cell line exhibits random XCI. Skewed XCI in undifferentiated hESCs may be due to clonal selection in culture instead of non-random XCI in ICM cells. We also found that *XIST* promoter methylation is correlated with silencing of *XIST* transcripts in early passages of hESCs, even in the pre-XCI state. In conclusion, XCI variations already take place in early passages of hESCs, which may be a consequence of *in vitro* culture selection during the derivation process. Nevertheless, we cannot rule out the possibility that XCI variations in hESCs may reflect heterogeneous XCI states in ICM cells that stochastically give rise to hESCs.

## Introduction

Human embryonic stem cells (hESCs) are an invaluable tool for regenerative medicine and a model for early human embryogenesis [1]. Numerous studies in the past ten years have described the capacity of hESCs to differentiate into specialized cells from the three germ layers [2]. In certain instances, *in vitro* differentiated hESCs can be integrated and become functional in transplantation experiments [3], [4]. Due to the wide applications of hESCs, there have been increasing demands for more newly derived hESC lines. This interest allows comparison of different properties among various hESC lines and can potentially create a gold standard for the characterization of hESC lines. Therefore, efforts have been made to generate gene expression and epigenetic profiles for hESCs [5], [6], [7], [8], [9]. Although it seems that the gene expression profile is quite consistent for all hESC lines, the epigenetic status varies significantly [6], [10]. For example, *XIST* gene expression varies among different hESC lines and even within the same cell line [5], [11], [12], [13].

In mice, *Xist* is known to play a major role in X chromosome inactivation (XCI) during female mammalian embryogenesis. In this process, genetic and epigenetic events, beginning with expression of *Xist*, allow equal expression from the X chromosome in male and female cells. In the mouse model system, XCI occurs in two waves during embryogenesis. At the two-cell stage, the imprinted paternal X chromosome is exclusively inactivated. During blastocyst formation, cells in the inner cell mass (ICM) reactivate the paternal X chromosome whereas the trophectoderm and primitive endoderm retain their imprinted XCI. Upon differentiation, the second wave of XCI occurs in a random fashion; thus each somatic cell will possess either maternal or paternal active X chromosome (reviewed in ref. [14]). Furthermore, it was shown that mouse ESCs (mESCs) and induced pluripotent stem cells (iPSCs) recapitulate random XCI upon differentiation [15], [16]. Due to prominent developmental differences between mouse and human, XCI patterns between the two species are not well conserved. Indeed, both random and skewed XCI patterns are observed in human extra-embryonic tissue (placenta), but not mouse extra-embryonic tissue, which show exclusive paternal X chromosome inactivation [17]. Therefore, an assessment of the XCI during early human embryogenesis is still needed.

A recent study in pre-implantation human embryos reported that *XIST* transcript accumulation on the X chromosome is initiated in the eight-cell stage embryo with full establishment of *XIST* clouds in the blastocyst stage [18]. However, the identity of the cells showing *XIST* accumulation is not obvious due to three distinct cell populations found in the blastocyst stage embryos, namely trophectoderm, primitive endoderm and ICM. Furthermore, the XCI pattern (skewed or random) is still unclear. Questions regarding the XCI status of the ICM and the pattern of XCI in human pre-implantation embryos still remain to be resolved.

Since differentiation of hESCs can be used to model human embryogenesis *in vitro*, they are examined for the initiation and maintenance of XCI. Several research groups including ours reported that XCI in hESCs, unlike their rodent counterparts, is unstable and prone to changes during culture [5], [10], [13], [19], [20]. Recent in depth XCI studies with several hESC lines described three different states of XCI in these cells [13], [19], [20] (reviewed by Dvash and Fan, 2009 [21]). The first or “naïve” state refers to undifferentiated hESCs that possess two active X chromosomes. However, upon differentiation, they acquire one inactivated X chromosome. The second or intermediate state refers to hESCs that show XCI markers in both undifferentiated and differentiated states. The third state describes hESCs that do not show any XCI markers in both undifferentiated and differentiated states. Interestingly, it has been suggested that hESCs progress gradually during culture from the first “naïve” state, to the second stage exhibiting XCI, then to the third state where all XCI marks are irreversibly lost [19] [21]. Furthermore, there is a correlation between the inability to express XCI marks and biallelic methylation pattern on the *XIST* promoter [13]. Importantly, all the above mentioned studies used mid to late passage hESCs (∼p20–p100), that have been exposed to long term culture effects.

It is therefore better to evaluate the status of XCI in early passages of undifferentiated hESCs that have been minimally exposed to culture effects. Hereby we report the status of XCI in ten lines of female hESC at the earliest passages available. Our results indicate that the three distinct states of XCI can be observed even in minimally passaged hESCs. In addition, we investigated the pattern of XCI in two cell lines- one showed random XCI reminiscent of mESCs, while the other showed non-random XCI. Consistently, we found that the methylation pattern of the *XIST* promoter is tightly associated with silencing of *XIST* expression in early passages of female hESCs.

## Results

### *XIST* expression analysis in CSES cell lines at early passages

Recent studies have identified three distinct states of XCI in a variety of female hESCs [12], [13], [19]. These studies have also implied that these three XCI states are the consequence of long term culture conditions. We hypothesized that by using early passage hESCs, which have minimal exposure to culture effects, we may be able to better evaluate XCI status in the derivation of hESCs. For this purpose, we used newly derived CSES cell lines [22] at the earliest available stage such as passage five (p5) for some of the cell lines to study XCI.

**Figure 1:**
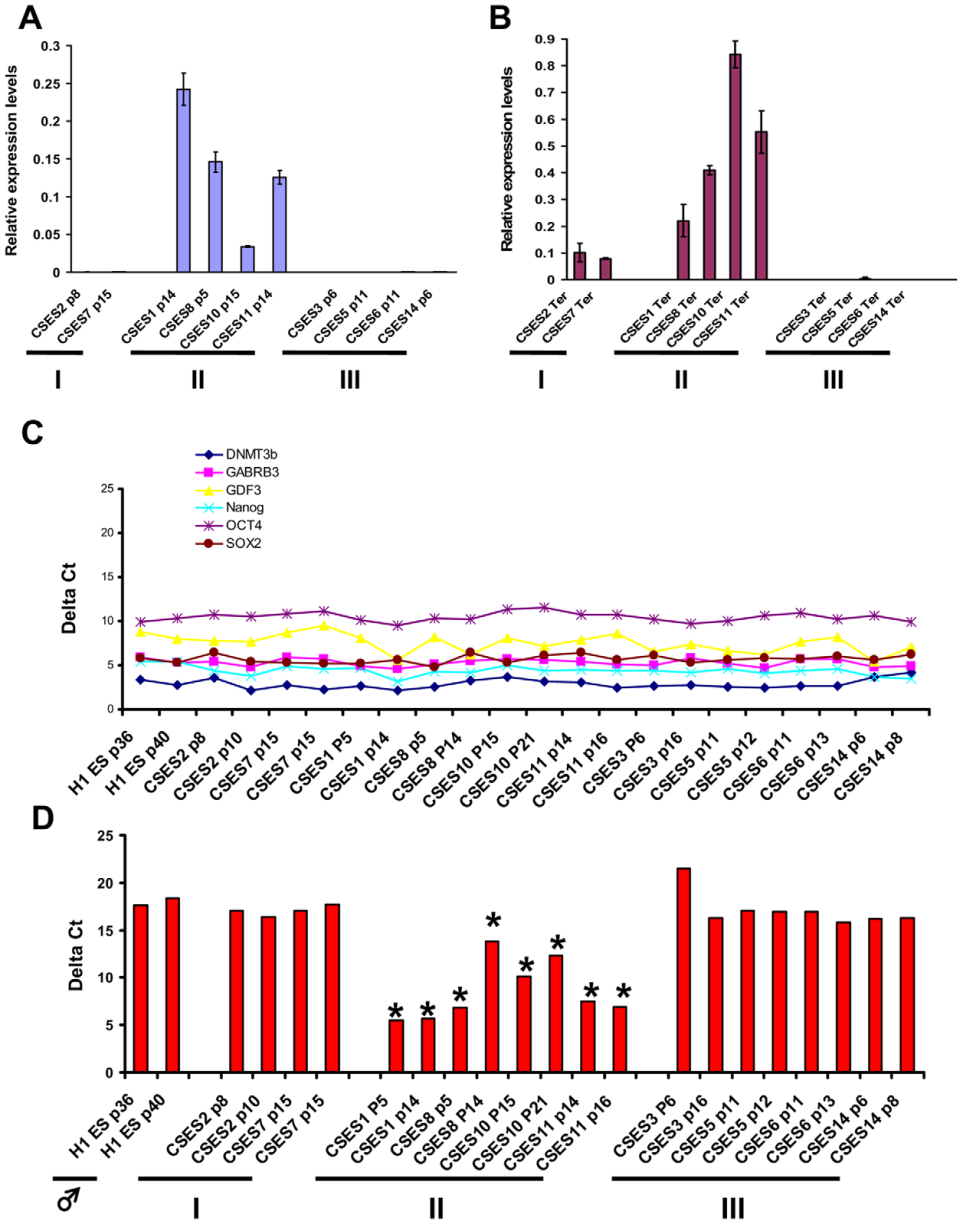
*XIST* expression in CSES cell lines. (A) *XIST* relative expression levels in CSES undifferentiated cells. (B) *XIST* relative expression levels in teratomas derived from CSES cells. (C) Pluripotency gene expression by Delta Ct in CSES cell lines in different passages as well as in H1 (male) cell line. (D) *XIST* expression by Delta Ct in all CSES cell lines analyzed compared to H1 (male) cell line. Significant values (p<0.01) are marked by asterisks.

Relative expression levels of *XIST* were assessed in all ten female cell lines (CSES1, 2, 3, 5, 6, 7, 8, 10, 11 and 14) by using real-time PCR analysis. In the undifferentiated state, four of the examined cell lines (CSES 1, 8, 10 and 11) expressed *XIST* while all the other lines did not show any *XIST* expression (Fig 1A). We then asked whether *XIST* expression can be induced in hESCs that do not express *XIST* upon teratoma differentiation. Consistent with previous findings [13] all cell lines that expressed *XIST* in the undifferentiated state were able to maintain and even up-regulate *XIST* expression upon differentiation. Interestingly, two of the cell lines (CSES2 and CSES7) that did not express *XIST* in the undifferentiated state were able to induce *XIST* expression upon differentiation, though at a lower level (Fig 1B). However, four cell lines (CSES3, CSES5, CSES6 and CSES14) did not express *XIST* both at the undifferentiated and differentiated state. These results clearly demonstrate that three different states of XCI exist in hESCs even after a short culture period. We conclude that CSES2 and CSES7 cells are in the “naïve” state, showing *XIST* expression only upon differentiation; CSES1, 8, 10 and 11 cells are in the intermediate state, showing *XIST* expression at the undifferentiated state and further induction upon differentiation; and CSES 3, 5, 6, 14 cells are in the third “culture affected” state, showing no *XIST* expression regardless of undifferentiated or differentiated conditions.

**Figure 2:**
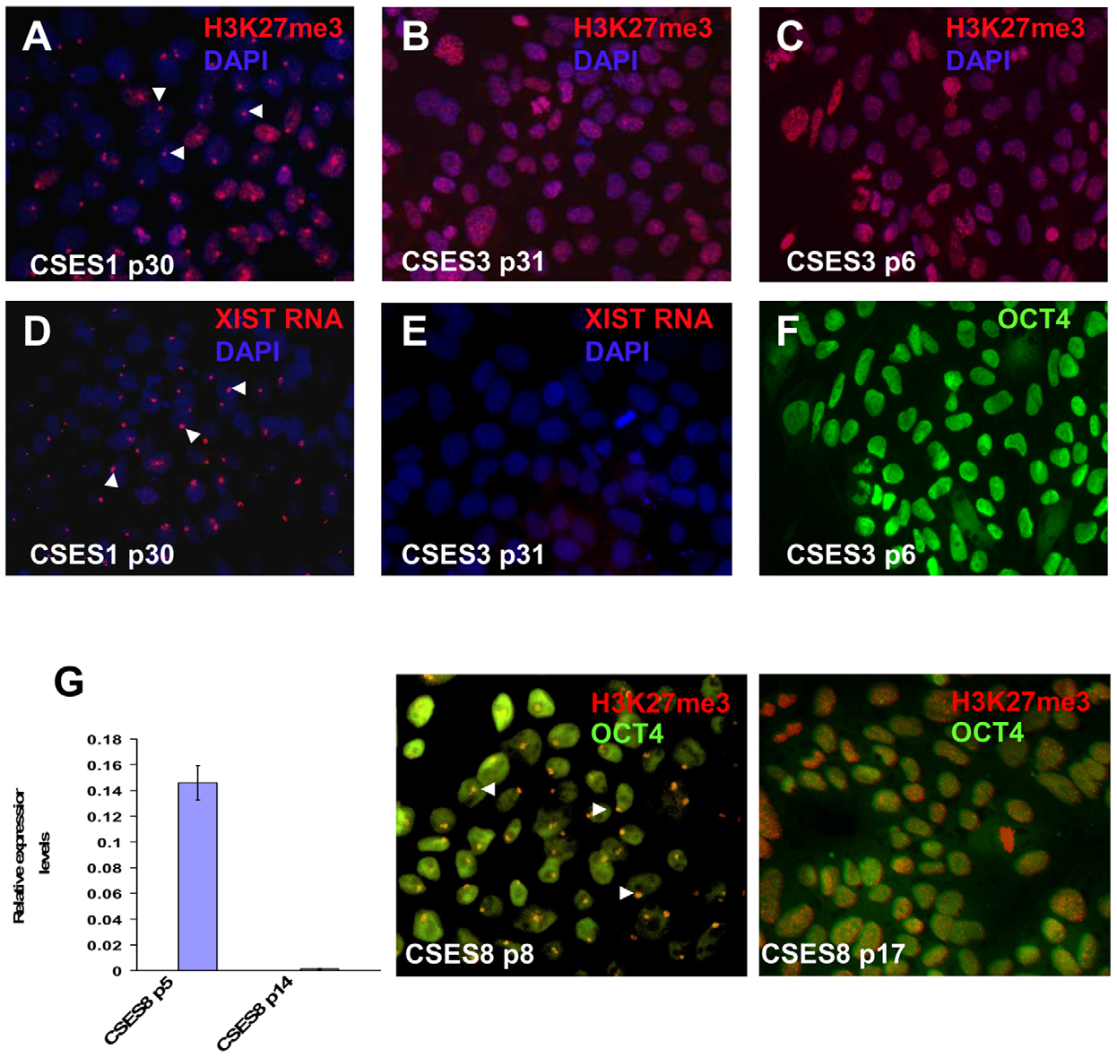
Stability of XCI markers in CSES lines. (A) CSES1 shows positive punctate staining for H3K27me3 (indicated by the arrow heads). CSES3 cells do not show punctate staining pattern for H3K27me3 in (B) late or (C) early passages. (D) Shown is *XIST* RNA coating of Xi by FISH in CSES1 cell line (indicated by the arrow heads). (E) CSES3 lack *XIST* RNA coating of Xi as indicated by FISH analysis. (F) CSES3 is positively stained for OCT4 (green) at p6. (G) CSES8 at p8 shows H3K27me3 punctate staining along with OCT4 staining; however, the relative expression of *XIST* is reduced in this cell line at p14. This observation is supported by a decrease in the cell population with H3K27me3 punctae in p17 (48% of the cells).

It has been shown that XCI initiation correlates with low levels of pluripotency related factors in mouse ES cells [23]. To exclude the possibility that *XIST* expression in undifferentiated hESCs occurs due to differentiation in culture, we analyzed pluripotency gene expression along with *XIST* expression by using the ABI human stem cell pluripotency low density array. Delta Ct values were calculated for each gene compared to the control gene, beta actin (Fig. 1C, D). Brown-Forsthye test shows that all pluripotency genes are expressed with small variance between different cell lines analyzed (p = 0.27) (Fig. 1C), whereas *XIST* expression varied significantly (p = 2.2×10^−17^) (Fig. 1D). These results were supported by our observation of simultaneous appearance of XCI markers and undifferentiated stem cell markers such as OCT4 by immunostaining (Fig. 2G).

To visualize XCI states at a single cell resolution, we assayed for *XIST* RNA coating of the inactive X (Xi) by using FISH analysis or enrichment of histone 3 lysine 27 tri-methylation (H3K27me3) by immunocytochemistry [24], [25]. Consistent with *XIST* expression, cell lines such as CSES1 showed punctate staining for H3K27me3 on the Xi (Fig. 2A). *XIST* RNA coating was also identified on one of the X chromosomes in virtually all of the cells (Fig. 2D). In contrast, cell lines such as CSES3 lack both H3K27me3 punctate staining and *XIST* RNA coating of the Xi (Fig. 2B, E). Interestingly, CSES3 lacks XCI markers at early passages (p6) (Fig. 2C, F) and upon long term culture (p31) (Fig. 2B, E). In addition, these cells lack the capability to induce XCI upon teratoma formation (Fig 1 B).

Low level of *XIST* expression as seen in CSES10 (Fig. 1A, D) can be explained either by uniformly low *XIST* expression in the entire cell population or by high expression from a small subset of cells. In order to distinguish between the two possibilities, we calculated the percentage of cells exhibiting punctate H3K27me3 staining. Indeed, all cell lines that do not express *XIST* also do not show any H3K27me3 punctate staining. However, in intermediate state (state II) cells, we observed a significant number of cells with punctate staining for H3K27me3 (Fig. S1). Therefore, low level of *XIST* expression in CSES10 p15 cells correlates with a lower number of H3K27me3 positive cells, indicating a low number of *XIST* expressing cells. Furthermore, all H3K27me3 positive cells were positively stained for OCT4 (data not shown), which again suggests that XCI can occur in undifferentiated hESCs.

Finally, we also observed the transition from the *XIST ^+^* to the *XIST ^−^* XCI state in subcultures of early passage hESCs. In CSES8, H3K27me3 punctate staining can be detected at a high percentage in p8 (n = 257, 87%); however the number of positively-stained cells was significantly reduced by p17 (n = 403, 48%). In parallel, *XIST* mRNA expression was dramatically reduced in p17 CSES8 hESCs (Fig 2G).

### Skewed versus random XCI pattern in CSES cell lines

During mouse embryogenesis, imprinted XCI of the paternal X chromosome occurs prior to the blastocyst formation whereas random XCI is initiated upon differentiation. However, previously we observed non-random XCI in later passages of female hESC lines [13]. Given that the XCI states seems to be affected by culture and that culture pressure could result in clonal selection, we were curious to see whether XCI is random or skewed in early passages of hESCs.

We chose to analyze the pattern of XCI in two cell lines, CSES1 and CSES8, because both showed XCI markers. In order to verify the parental origin of the active X chromosome, we used maternal DNA that was extracted from granulosa cells, which are somatic cells that surround the oocyte during maturation. These cells are normally removed at the time of egg retrieval during IVF procedure in order to expose the oocyte surface for fertilization. Frozen granulosa cells for CSES1 and CSES8 were used for DNA extraction. Following DNA amplification these samples were hybridized to Affymetrix SNP array (250k Sty) side by side with the corresponding hESC DNA. The results from this analysis enabled us to choose SNPs within X-linked genes that were identified as heterozygous in the hESC lines and homozygous in the maternal DNA.

In order to verify the maternity of the granulosa DNA sample with the corresponding hESC sample, we performed identical by state (IBS) analysis to show that our samples are genetically related. For instance, comparison of CSES1 and its corresponding maternal sample showed that in 71.7% of the SNPs both alleles are shared (IBS = 2) and in 28% of the SNPs one of the alleles is shared (IBS = 1). This indicates that 99.7% of SNPs show at least one allele shared between the samples. Overall 85.7% of SNPs are shared between CSES1 and its maternal sample. This indicates close genetic relationship between the two samples (Fig. S2). In order to perform SNP expression analysis, we selected genes that show moderate to high expression in undifferentiated hESCs. Consequently, we analyzed the expression of seven SNPs for CSES1 and nine SNPs for CSES8 along the X chromosome (Table 1, 2, Fig. S3, S4).

**Table 1:** Genomic SNP genotyping and polymorphic cDNA analysis of CSES1.

SNP ID	Band	chromosome location	Gene name	CSES1 genotype	Granulosa genotype	CSES1 p5 expression	CSES1 p14 expression
rs5914796	Xp11.21	56807583	DKFZp686L07201	A/T	T/T	A	A
rs4828327	Xq21.1	84236784	SATL1	A/C	C/C	A	-
rs6620161	Xq21.33	96027150	DIHPA2	A/G	G/G	A/G	A/G
rs2428212	Xq24	118985598	UPF3B	A/G	G/G	A	A
rs6641482	Xq28	147887801	AFF2	G/A	A/A	G	-
rs41537046	Xq26.2	132470683	GPC4	A/G	G/G	A	A/G
rs895744	Xq28	153998985	BRCC3	G/T	T/T	G	-

**Table 2:** Genomic SNP genotyping and polymorphic cDNA analysis of CSES8.

SNP ID	Band	Chromosome location	Gene name	CSES8 genotype	Granulosa genotype	CSES8 p5 expression	CSES8 p14 expression
rs3747276	Xp22.11	21985464	SMS	A/G	G/G	A/G	A/G
rs6628886	Xp21.1	34654777	TMEM47	A/G	G/G	A/G	A/G
rs6625472	Xq13.1	68739542	TMEM28	A/G	G/G	A/G	A/G
rs479640	Xq13.2	73668829	SLC16A2	C/T	T/T	C/T	C/T
rs717689	Xq21.1	77379935	PGK1	A/G	G/G	A/G	A/G
rs1204399	Xq22.1	99886830	TSPAN6	A/G	G/G	A/G	A/G
rs2294504	Xq23	109552667	AMMECR1	C/T	T/T	C/T	C/T
rs42890	Xq24	119578513	LAPM2	T/G	G/G	T/G	T/G
rs5977910	Xq26.2	132901293	GPC3	T/G	T/T	T/G	T/G

Our results indicate discrepancies between the two hESC lines with regard to the pattern of XCI. In the CSES8 cell line at both p5 and p14, we consistently observed bi-allelic expression of X-linked genes, indicating that random XCI has occurred. On the other hand, CSES1 at p5 showed preferential expression of the paternal allele suggesting skewed XCI with six out seven X-linked genes exhibiting mono-allelic expression from the paternal X. Theoretically, the probability of having maternal or paternal allele expressed per SNP is 50%. According to binomial distribution B(x = 6; n = 7, p = 0.5), the cumulative probability of getting as many as six out of seven SNPs with paternal expression is 0.0625. Since this probability is very small, it argues against the hypothesis of random XCI in this case. Therefore, the preferential expression from the paternal allele is due to skewed maternal X inactivation. Moreover, upon prolonged culture of CSES1 cell line, we observe partial reactivation of the second allele that coincides with the loss of *XIST* expression at later passages. For example, rs41537046 expressed only the paternal allele (A) at p5, whereas both paternal and maternal alleles are expressed (A/G) by p14 (Table 1 and Fig. S3). This is consistent with our previous observation that loss of *XIST* expression would lead to partial reactivation of a portion of previously silenced genes in Xi [13].

### *XIST* promoter methylation status correlates with the three different states of XCI in hESCs

**Figure 3:**
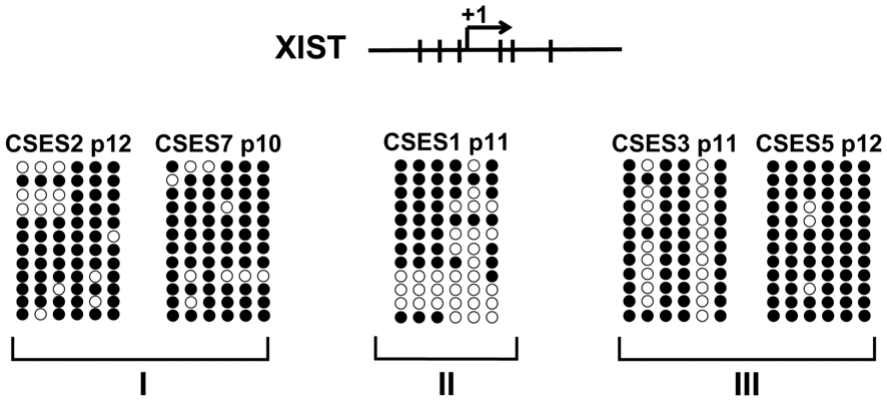
*XIST* promoter methylation analysis in CSES cell lines. Schematic of *XIST* promoter CpG sites and bisulfite sequencing results of the corresponding CpG sites in the *XIST* promoter in each cell line. Open circle represents unmethylated CpG site and closed circle represents methylated CpG site.

The variability of XCI status in early passages may indicate epigenetic modifications of the *XIST* promoter that occur either prior or subsequent to the hESCs derivation process. Indeed, it was previously suggested that *XIST* promoter methylation pattern is correlated with the XCI status of the cells [13], [21]. Consistently, our analysis demonstrates that CSES1 at p11 expresses XCI markers and have ∼50% methylation at the *XIST* promoter (Fig. 3). This result is consistent with the observation that one of the *XIST* alleles in these cells expresses *XIST* in the undifferentiated state as well as upon teratomas differentiation. In addition, the *XIST* promoter shows ∼90% methylation in later passages of CSES8 (p28) consistent with silencing of both *XIST* alleles and the loss of XCI markers upon long-term culture (data not shown). We also observed 70–96% methylation in the *XIST* promoter of CSES3 and CSES5 (Fig. 3), even in early passages (p11 and p12 respectively). This is correlated with the inability of these cell lines to induce XCI in either undifferentiated state or upon teratomas differentiation (Fig. 1B). Interestingly, both CSES2 and CSES7, which exhibit XCI only upon differentiation, also showed 80–87% methylation of the *XIST* promoter in early passages (p12 and p10 respectively) (Fig. 1B and Fig. S5). This observation suggests that DNA methylation is also involved in repression of *XIST* gene expression in the pre-XCI state.

## Discussion

In this study, we attempted to minimize the *in vitro* culture effects by using hESCs at the earliest available time. It has been shown that long term culture can contribute to the variation in XCI status of hESCs [13], [19], [20]. We found that early passage cell lines already exhibit various XCI states similar to cell lines after prolonged culture. While it is formally possible that culture variations in the first few weeks of hESC derivation and expansion may yield XCI variation, we cannot rule out the alternative possibility that XCI variations may reflect innate heterogeneity of the original cells found in the ICM of the human embryo. This hypothesis is supported by a study on cultured mouse ICM cells reporting that these cells can undertake different paths- such as differentiation to epiblast, reversion into pre-blastocyst embryonic cell stage or selective expansion of a distinct subpopulation- and therefore differ in their XCI status [30]. Of note, mouse EpiSCs exhibit XCI as they are derived from a later stage of mouse embryogenesis and resemble the morphology and the expression patterns of the hESCs [28], [29], [32]. Thus, it is formally possible that at the time of the hESCs derivation, some of ICM cells have already progressed to a more committed state and already initiate XCI [24], whereas other ICM cells are still at a pre-XCI stage that eventually give rise of hESCs in the “naïve” pre-XCI state.

Here we show that variations of XCI are already present from early passage (p5) to later passage (p14) of the same cell line (CSES8), supporting the notion that XCI is highly affected by culture conditions [13], even at the earliest stages of culturing. Very recently, it was demonstrated that derivation of hESCs in physiological oxygen concentration (5%) allows establishment of hESC in a naïve pre-XCI state, with two active X chromosome [31]. Irreversible XCI occurs when the naïve female hESCs are cultured in atmospheric oxygen concentration (20%) conditions. This is consistent with the previous notions that female hESCs can switch from a pre-XCI state to established XCI status upon culture selection.

Concerning the pattern of XCI in hESCs at the early passages, our SNP analysis revealed random XCI for one line of hESCs (CSES8) while skewed XCI for the other cell line (CSES1) with preferential expression from the paternal X chromosome. Skewed XCI is known to happen during mouse embryogenesis at the two cell stage until the blastocyst stage when the inactive paternal X becomes reactivated. In this case, the inactivation is imprinted and the paternal X chromosome is inactivated. In hESCs, methylation specific analysis of a polymorphic tri-nucleotide repeat at the *HUMARA* gene show three out of four hESC lines possess skewed XCI while one has a random pattern of inactivation [33]. Interestingly, this research group also analyzed a triploid (3PN) hESC line and showed a progression from a random XCI to a skewed pattern of XCI. Similarly, transition of naïve hESCs, possessing two active X chromosomes under physiological oxygen concentrations to atmospheric oxygen concentrations also result in skewed XCI [31]. We show that in CSES1 cells the maternal X chromosome is inactivated. Our observations for CSES1 cell line combined with the observation from Liu et al. [33] and Lengner et al. [31] suggest that culture selection during hESC culture may results in skewed XCI as reported [13], [31], [33].

The mechanism underlying the initiation and loss of XCI markers in hESCs is largely unknown. It is known that *Xist* promoter is 50% methylated in undifferentiated female mESCs [34]. However, the methylation status of human *XIST* promoter in the pre-XCI state is previously unknown. In order to gain some insight into this mechanism, we analyzed the methylation patterns of *XIST* promoter in early passages of hESCs. Cells that show XCI markers both prior and post differentiation have approximately 50% methylation of the *XIST* promoter as expected. We also observe gain of methylation in the *XIST* promoter region upon further passages, which is also associated with loss of *XIST* expression and other XCI markers in the case of CESE8 hESCs. Here we show that hypermethylation of the *XIST* promoter is also observed in short term culture (CSES3 and CSES5). This correlates with the inability of these cells to initiate XCI upon differentiation. Surprisingly, cell lines such as CSES2 and CSES7 were able to initiate XCI upon differentiation but showed relative high methylation on the *XIST* promoter in the undifferentiated state, consistent with reports by Lengner et al. [31] for their hESCs in the pre-XCI state. The ability of these two CSES cell lines to initiate *XIST* expression upon differentiation suggests two possibilities: (i) there might be an additional XCI state in hESC where hypermethylation of the *XIST* promoter is reversed and consequently *XIST* transcript is expressed, or (ii) only a minority of the cells within the teratomas express *XIST* whereas the majority of cells do not express *XIST*.

In this study, we propose that XCI occurs in undifferentiated hESC in a random manner and the observations of skewed XCI are probably a result of a clonal selection occurring in hESC culture. It was recently shown that mouse induced pluripotent cells (miPSC) recapitulate XCI patterns of mESCs [16]. Therefore, analysis of human iPSCs may elucidate whether these cells can reactivate both X chromosomes and maintain their active state in the undifferentiated state, or whether XCI occur in these cells in the undifferentiated state as in hESCs. Moreover, previous reports have demonstrated the involvement of pluripotency factors in the initiation of XCI in mESCs [23]. Since hESCs and mEpiSCs seems to represent a more committed derivative of the ICM it is possible that a different set or levels of transcription factors present in these cells are responsible for the different patterns of XCI between mouse and human ESCs.

Overall the variability in the status of XCI among the cell lines indicates either rapid epigenetic culture effect or the potential heterogeneity of the original ICM cells. It seems that the cells, even in early passages, tend to undergo XCI and later on lose the XCI markers. Thus, XCI process is highly affected by culture conditions and inactivation of one of the X chromosomes may provide an advantage in the current culture condition. We propose that a careful re-examination of XCI status in human ICM will shed light on the status and pattern of XCI in these cells. It is known that normal XCI is critical in the embryonic development [35] and inappropriate XCI is involved in different pathologies such as cancer [36]. Therefore, routine evaluation of XCI status should be a standard procedure for any pluripotent cells, including iPSCs, when they are applied to regenerative medicine.

## Materials and Methods

### Ethic statement

This research was approved by UCLA Embryonic stem cells research oversight (ESCRO) committee.

### Human ESC culture and differentiation

Cedars Sinai Embryonic Stem cell lines (CSES) were derived as described [22] and were cultured on mouse embryonic fibroblasts (MEF) in hESC medium [2] supplemented with 30ng/ml bFGF until cell line establishment. Upon establishment bFGF concentration was reduced to 5ng/ml. Manual passaging method was applied until a stable cell line was established (normally between p4 to p8), and thereafter enzymatic transfers were done either by using Trypsin/EDTA or collagenase type IV [2]. Cells were allowed to undergo *in vivo* differentiation by injection of 5×10^6^ cells under the kidney capsule of Nude mice. A month after injection, the mice were euthanized and teratomas were removed for RNA extraction. The care of the animals was in accordance with the institutional guidelines, as approved by Cedars Sinai Medical Center Institutional Animal Care and Use Committee, according to protocol 2182. *In vitro* differentiation was carried out on matrigel coated plates and the cells were cultured in hESC medium without bFGF supplemented with 2 µM retinoic acid (Sigma) for seven days.

### RNA extraction and real time polymerase chain reaction analysis

Total RNA was extracted using RNeasy mini kit (Qiagen, Valencia, CA http://www1.qiagen.com) and was treated with DNase I as described in the RNeasy mini kit protocol. 1 µg of DNase treated RNA was subjected to reverse transcription by iScript cDNA synthesis kit (Bio-Rad, Hercules, CA http://www.bio-rad.com). cDNA was either subjected to PCR amplification for SNP analysis as described below or for real-time PCR that was carried out with Bio-Rad iCycler using IQ™ SYBR® green supermix (Bio-rad) with *XIST* and *GAPDH* human specific primers (see table S1 for primer sequences). Relative *XIST* gene expression levels were calculated after they were normalized with expression levels of GAPDH.

### Gene expression analysis by low density arrays

RNA from undifferentiated hESCs were analyzed by a Taqman© based assay, using the human stem cell pluripotency array (ABI, foster city,CA). Delta Ct values were obtained by identifying the number of amplification cycles needed to reach the common threshold (Ct) value for each gene. Then, these values were normalized by subtraction of the Ct values obtained for a control gene (beta-Actin) for the same sample. In order to show that *XIST* expression variation among the different cell lines is not due to differentiation in culture, we performed a Brown-Forsthye test.

### SNP analysis

Genomic DNA from the hESC lines was extracted using DNeasy kit (Qiagene). Due to low amount of starting cells, DNA for Granulosa cells was extracted with the same kit and was subjected to whole genome amplification using REPLI-g mini kit (Qiagene). DNA from CSES1 (p14), CSES8 (p12) and their corresponding maternal granulosa cells were hybridized to Affymetrix 250k Sty SNP array (Affymetrix, Santa Clara, CA http://Affymmetirx.com). The microarray data have been deposited in GEO and given the series accession number GSE2167. SNPs were selected for analysis along the X chromosome according to the following criteria: the gene is X-linked and expressed in hESCs in moderate to high levels, and the SNP is heterozygous in the hESC line and homozygous in the corresponding maternal DNA. Genotype was validated for the selected SNPs by direct sequencing (see table S1 for primer sequences). In order to assess the expression from a specific SNP in CSES1 and CSES8 hESC lines, we used cDNA that was treated with DNase I as described above to avoid DNA contamination and amplified the specific regions. Amplicons were separated using an ethidium bromide stained 2% agarose gel, followed by gel-purification (Wizard ® SV Gel and PCR clean up system, Promega http://www.promega.com) and direct sequencing reaction to identify the expressed allele.

### Bisulfite genomic sequencing analysis

Genomic DNA samples for CSES1, 2, 3, 5 and 7 were digested with *Bgl*II overnight and treated the following day with sodium bisulfite for 15 hours as previously described [37]. Converted DNA samples were cleaned using Wizard® DNA Clean Up Kit (Promega) and were amplified by a single PCR reaction. PCR products were cloned into the Topo TA Vector 4.0 (Invitrogen). Individual colonies were picked for sequencing to identify the allelic methylation patterns. *XIST* promoter Bisulfite PCR primers were designed using the MethPrimer online software http://www.urogene.org/methprimer/index1.html.

### Immunocytochemistry and *XIST* RNA-FISH analysis

Cells for immunostaining were first fixed with 4% PFA/PBS for 20 minutes at room temperature and washed with PBS. Cells were then permeabilized by 0.4% Triton-X in TBST for 20 min and blocked in 10% milk and 1% normal goat serum for 1 hour. Cover slips were incubated 1 hour at room temperature with primary antibodies diluted in 3% BSA in TBST [monoclonal mouse anti-OCT4 (1∶20, Santa Cruz) and polyclonal rabbit anti-H3K27me3 (1∶1000, a gift from Yi Zhang, University of North Carolina, Chapel Hill, NC)]. After being washed three times with PBS, cover slips were incubated in fluorochrome-conjugated secondary antibodies for 1 hour at room temperature with protection from light. Hoechst dye #33342 was used to label cell nuclei. *XIST* RNA-FISH was performed as described [38] by using three 50-mer DNA probes designed from consensus sequences of map positions 6183-6232, 62234-6283 and 6368-6417 (accession No. L04961), which are in repeat D of *XIST*.

## Supporting Information

Table S1 Primer sequences.
(0.05 MB DOC)

Figure S1 Percentage of undifferentiated cells positively stained for H3K27me3. Intermediate state (state II) cells show significant number of cells with punctate staining for H3K27me3 CSES1 p15 (n = 511, 94%), CSES8 p8 (n = 257, 87%) CSES10 p15 (n = 257, 32%) and CSES11 p12 (n = 655, 84%).
(4.55 MB TIF)

Figure S2 Identical by state (IBS) analysis for CSES1 and its granulosa cells. Proportion of IBS = 0, 0.3% (no shared alleles), IBS = 1, 28% (one shared allele) and IBS = 2, 71.7% (two shared alleles). Overall, 85.7% of the alleles are shared, clearly indicating for close genetic relationship between the samples.
(8.34 MB TIF)

Figure S3 SNP sequences for CSES1 samples. SNP rs6620161 shows biallelic expression in p5 with one of the alleles more prominently expressed. However, in p14 both alleles are expressed at the same level. rs41537046 shows monoallelic expression at p5, but at p14 both of the alleles are already expressed. SNP rs5914796 shows expression of the paternal allele both at p5 and p14.
(9.12 MB TIF)

Figure S4 SNP sequences for CSES8 samples. Representing SNP sequences for CSES8 cell line. SNPs rs1204399, rs42890 and rs6625472 all show biallelic expression both at p5 and p14.
(9.24 MB TIF)

Figure S5 Induction of XCI in CSES upon Retinoic Acid differentiation. XCI detected by immunostaining for H3K27me3 and pluripotency detected by staining for OCT4 were tested in the three different classes of CSES cells. In CSES2 and CSES7 (A–D), representing class I cells, we were able to detect induction of XCI upon differentiation in CSES2 (A, B) but not for CSES7 (C, D). CSES3 representing class II cells were not able to induce XCI upon differentiation (E, F). In CSES8, we were able to detect XCI markers both in the undifferentiated and differentiated cells (G, H).
(9.89 MB TIF)
