# Supplementary material for: Variations of X Chromosome Inactivation Occur in Early Passages of Female Human Embryonic Stem Cells
Source: PLoS One. 2010 Jun 25;5(6):e11330. doi: 10.1371/journal.pone.0011330 (PMC2892515; doi:10.1371/journal.pone.0011330)
Supplement: Table S1 — Primer sequences. (0.05 MB DOC) [file pone.0011330.s001.doc]

**Supplementary table 1 – Primer sequences**

| **Gene/SNP name** | **F primer** | **R primer** | **Tm** | **Product length (bp)** |
| --- | --- | --- | --- | --- |
| XIST Real Time | atgcctggcactctagcact | gcaagagaaacatggaaatgg | 60 | 125 |
| GAPDH Real Time | atggggaaggtgaaggtcg | ggggtcattgatggcaacaata | 60 | 108 |
| XIST BS | ttgtttttattgggtaaattttgaatta | aaaaccccaaatacaaaaaaatctt | 60 | 204 |
| rs5914796 | gaacgcaggaactttggtgt | aatgaacaggagggaacgtg | 60 | 245 |
| rs6620161 | attttgggcccttttctcat | aggcggcctttttcttttta | 60 | 160 |
| rs4828327 | tgctgagtgttgggtagctg | aggagatggtggataggaagc | 60 | 203 |
| rs2428212 | ggaaaggggtaaatgaaaaagg | ctctaggaagcaacgcaatg | 60 | 233 |
| rs6641482 | cctggaaaggcatgctgtat | gccaggcctccaactaaag | 60 | 220 |
| rs41537046 | tggaatgctggaatactgga | acaacagagcaaggctccat | 60 | 195 |
| rs895744 | ggcaaaggaacaaaaagctg | tgagaccgaaattatgggaca | 60 | 245 |
| rs3747276 | ccttcttccccttgttctcc | caccaagttccattcccatc | 60 | 157 |
| rs6628886 | agcaacacctgaaggcattt | gaagagaacgtggcaagagg | 60 | 246 |
| rs6625472 | ttctaggacaggagggctga | agccaaacatccccaatttt | 60 | 184 |
| rs479640 | ttgatctcaggggaatttgg | cctggctccaagaacagaag | 60 | 246 |
| rs717689 | ttgaataagccctcctggtg | catctagtggccaaggatgc | 60 | 151 |
| rs1204399 | caaaactctcagttactggtcaca | gggcactgagtctttccaca | 60 | 250 |
| rs2294504 | cattgttacccaaagccaca | tgcagcaatccaagatctca | 60 | 226 |
| rs42890 | ttatgtgcaggctgctatgg | gtctggcactcatcccattt | 60 | 202 |
| rs5977910 | catgactctaaggcaacacagg | cagctgtttggctacactgg | 60 | 160 |
