# Supplementary figures and images for: Variations of X Chromosome Inactivation Occur in Early Passages of Female Human Embryonic Stem Cells
Source: PLoS One. 2010 Jun 25;5(6):e11330. doi: 10.1371/journal.pone.0011330 (PMC2892515; doi:10.1371/journal.pone.0011330)

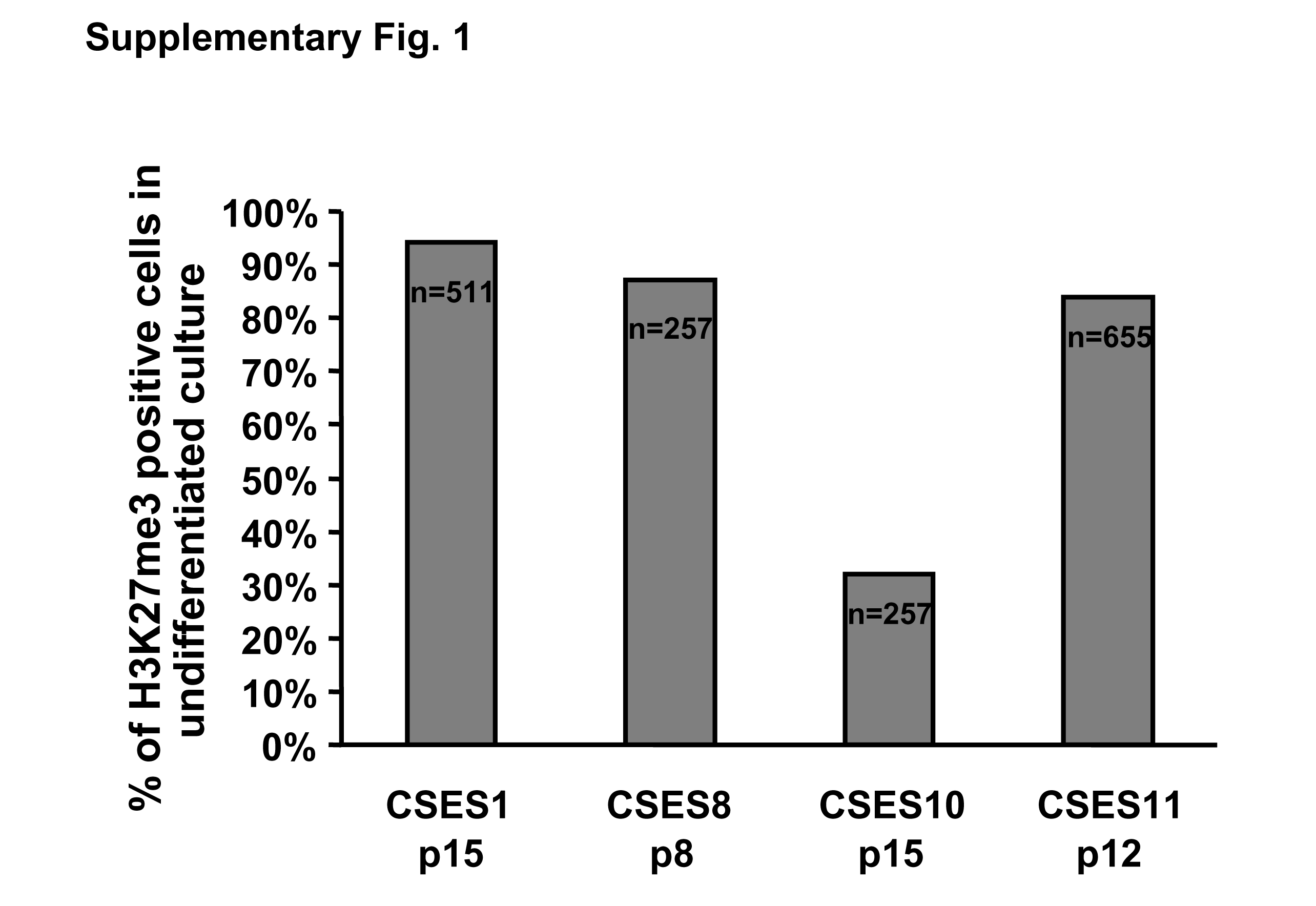

Supplement: Figure S1 — Percentage of undifferentiated cells positively stained for H3K27me3. Intermediate state (state II) cells show significant number of cells with punctate staining for H3K27me3 CSES1 p15 (n = 511, 94%), CSES8 p8 (n = 257, 87%) CSES10 p15 (n = 257, 32%) and CSES11 p12 (n = 655, 84%). (4.55 MB TIF) [file pone.0011330.s002.tif]

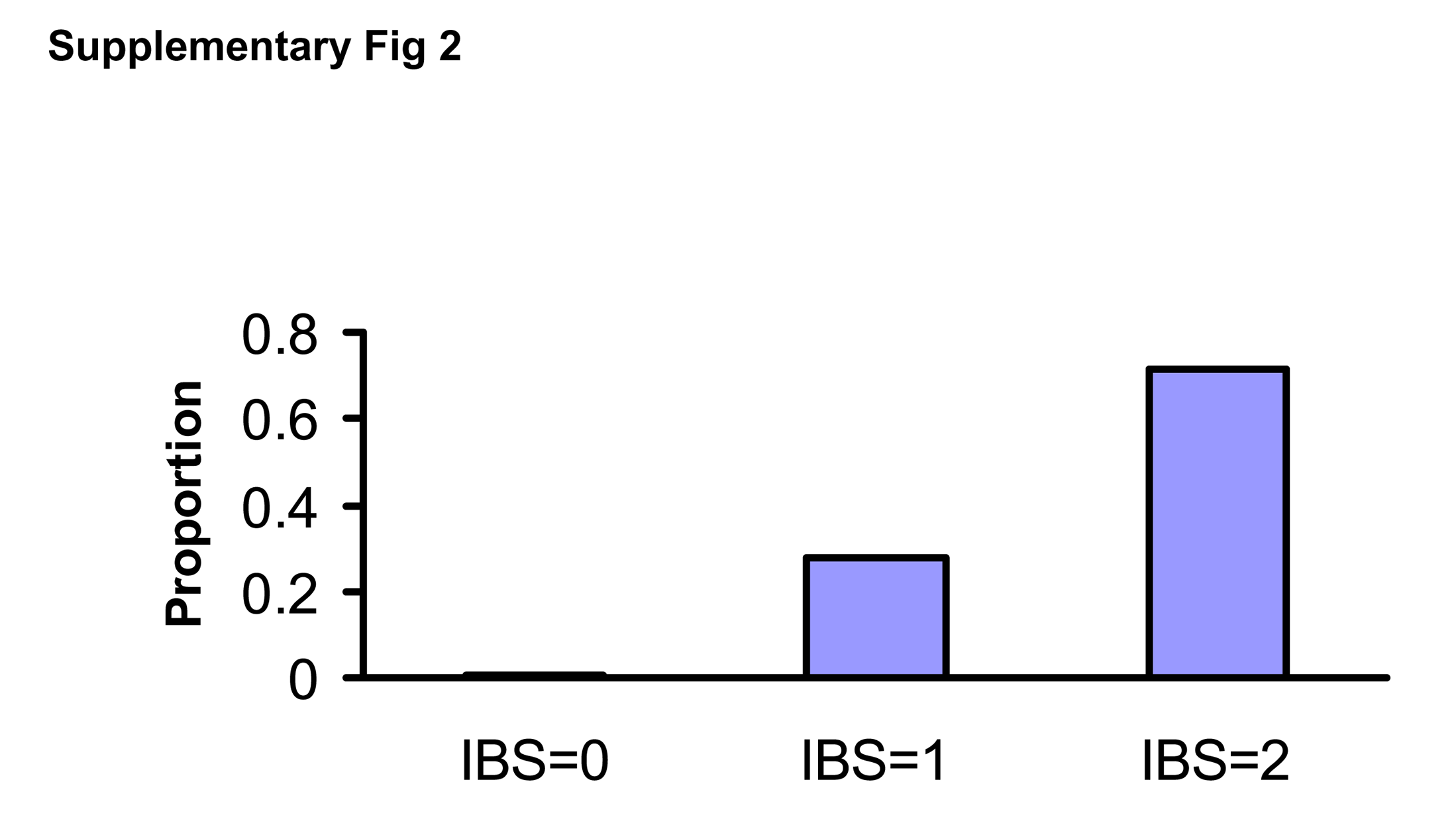

Supplement: Figure S2 — Identical by state (IBS) analysis for CSES1 and its granulosa cells. Proportion of IBS = 0, 0.3% (no shared alleles), IBS = 1, 28% (one shared allele) and IBS = 2, 71.7% (two shared alleles). Overall, 85.7% of the alleles are shared, clearly indicating for close genetic relationship between the samples. (8.34 MB TIF) [file pone.0011330.s003.tif]

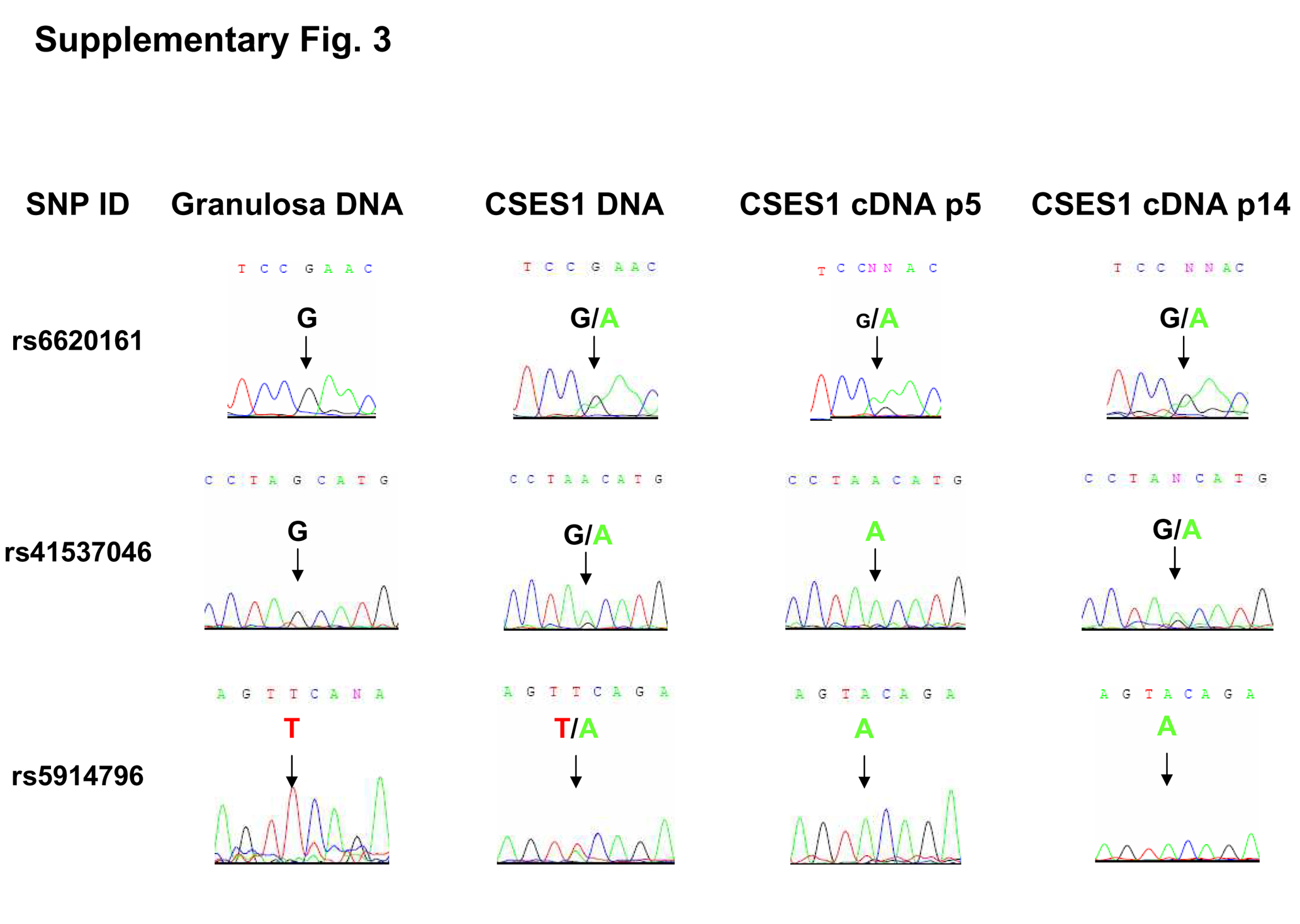

Supplement: Figure S3 — SNP sequences for CSES1 samples. SNP rs6620161 shows biallelic expression in p5 with one of the alleles more prominently expressed. However, in p14 both alleles are expressed at the same level. rs41537046 shows monoallelic expression at p5, but at p14 both of the alleles are already expressed. SNP rs5914796 shows expression of the paternal allele both at p5 and p14. (9.12 MB TIF) [file pone.0011330.s004.tif]

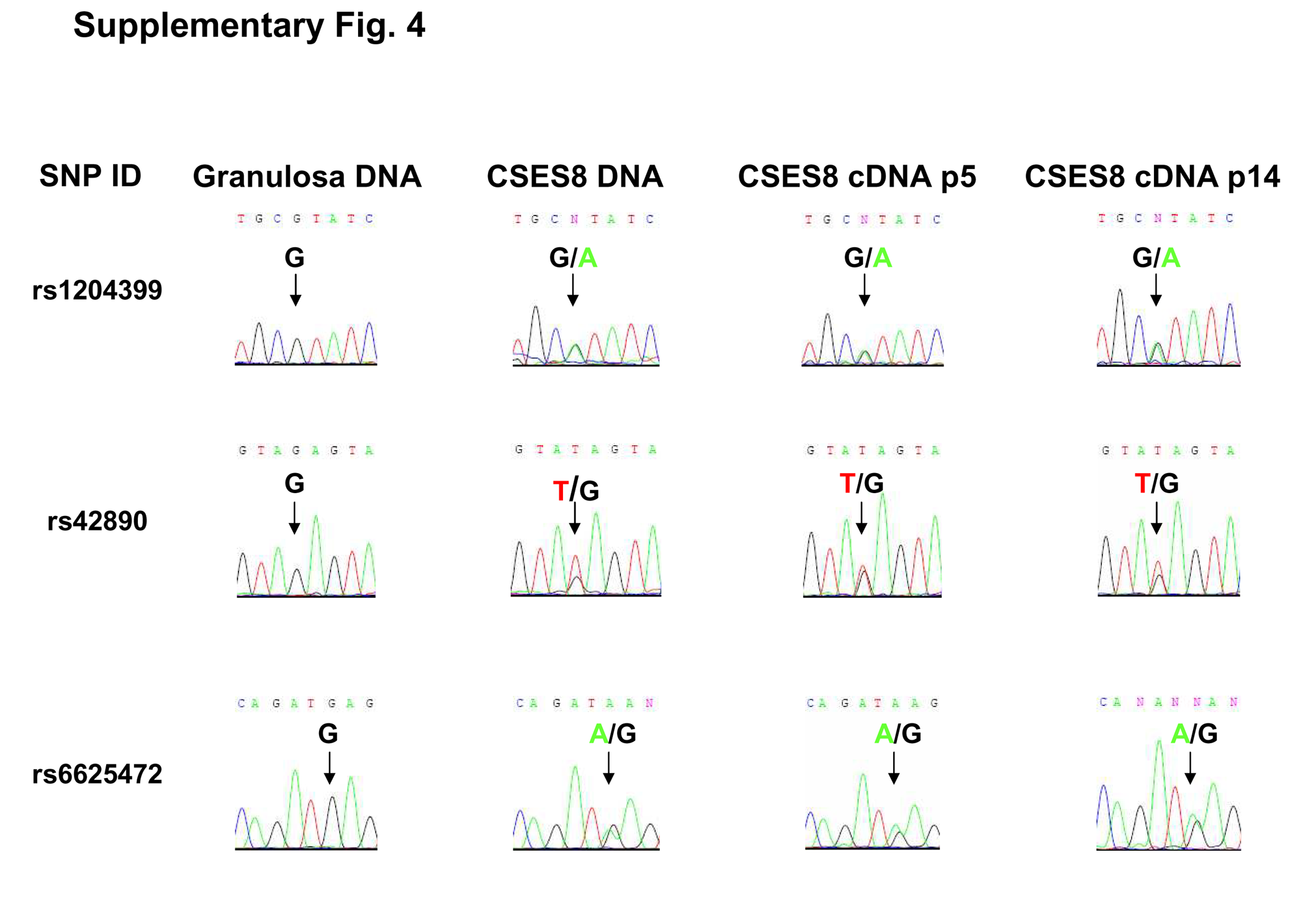

Supplement: Figure S4 — SNP sequences for CSES8 samples. Representing SNP sequences for CSES8 cell line. SNPs rs1204399, rs42890 and rs6625472 all show biallelic expression both at p5 and p14. (9.24 MB TIF) [file pone.0011330.s005.tif]

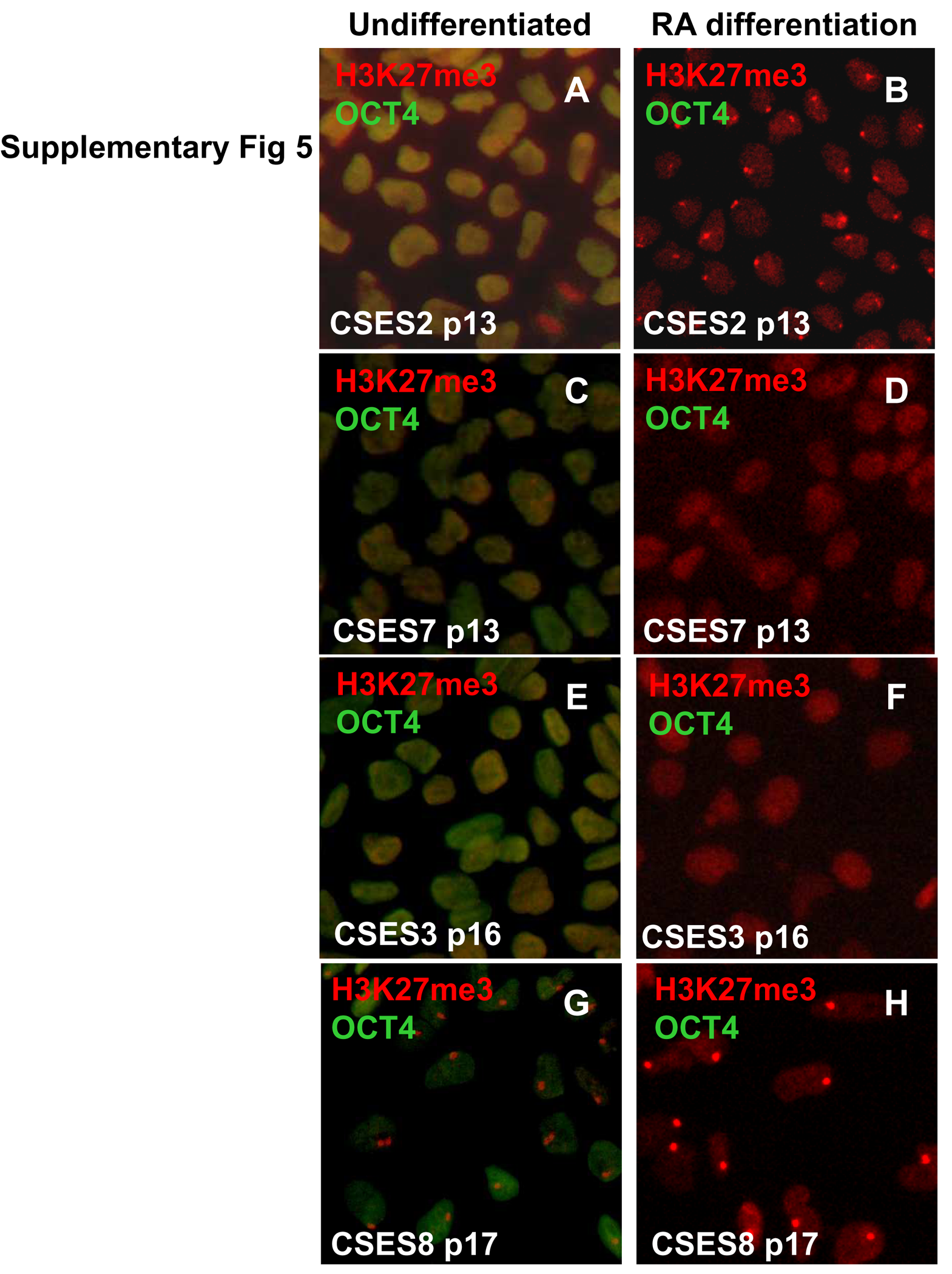

Supplement: Figure S5 — Induction of XCI in CSES upon Retinoic Acid differentiation. XCI detected by immunostaining for H3K27me3 and pluripotency detected by staining for OCT4 were tested in the three different classes of CSES cells. In CSES2 and CSES7 (A–D), representing class I cells, we were able to detect induction of XCI upon differentiation in CSES2 (A, B) but not for CSES7 (C, D). CSES3 representing class II cells were not able to induce XCI upon differentiation (E, F). In CSES8, we were able to detect XCI markers both in the undifferentiated and differentiated cells (G, H). (9.89 MB TIF) [file pone.0011330.s006.tif]
